# Impact of early-onset peritonitis on mortality and technique survival in peritoneal dialysis patients

**DOI:** 10.1186/s40064-016-3369-9

**Published:** 2016-09-29

**Authors:** Sheng Feng, Yancai Wang, Beifen Qiu, Zhi Wang, Linseng Jiang, Zhoubing Zhan, Shan Jiang, Huaying Shen

**Affiliations:** Department of Nephrology, Second Affiliated Hospital of Soochow University, 1055 Sanxiang Road, Jinchang, Suzhou, 215000 Jiangsu China

**Keywords:** Early onset peritonitis, Mortality, Peritoneal dialysis

## Abstract

**Background:**

Early onset peritonitis (EOP) is not uncommon in peritoneal dialysis patients. We aimed to compare the prognosis of EOP and non-EOP peritoneal dialysis patients.

**Methods:**

This study included subjects that underwent PD from January 1, 2004 to July 31, 2013. Patient characteristics were collected. EOP was defined as peritonitis occurring within 6 months after initiation of PD. Patient and technique survival were compared between EOP and non-EOP patients using Cox regression analyses.

**Results:**

In total, 189 subjects were included in this study. Patients were divided into EOP (n = 55) and non-EOP groups (n = 134). There was no significant difference in the causative organisms of peritonitis between the two groups. After adjusting for age, diabetes status, serum albumin level and residual renal function, the multivariable Cox regression model revealed that EOP was an independent risk factor for patient mortality (HR 2.03, RI 1.09–3.80, p = 0.026), technique failure (HR 1.69, RI 1.12–2.87, p = 0.015) and total survival (HR 1.73, RI 1.12–2.68, p = 0.013).

**Conclusions:**

EOP was identified as an independent risk factor for mortality and technique failure in peritoneal dialysis patients.

## Background

Peritoneal dialysis (PD) is a well-established treatment for end-stage renal disease (Li and Chow 2013; Mujais and Story 2006; Pecoits-Filho et al. 2007). Due to improvements in connectology, peritonitis, a common and serious complication of PD, has decreased dramatically (Daly et al. 2001, 2014). Over the past several decades, the role of peritonitis as an independent risk factor for mortality and technique failure in PD patients has been well established (Brown et al. 2007; Davenport 2009; Fried et al. 1996; Kavanagh et al. 2004; Mizuno et al. 2011). However, recent studies on this topic have shown contradictory results (Fang et al. 2008; Isla et al. 2014).

Peritonitis occurs more frequently in newly initiated PD patients because of unskilled PD manipulation. In the BRZPD study (Martin et al. 2011), the median time from PD initiation to first peritonitis episode was found to be 6 months. Another study also showed that during the first year after PD, more than 70 % of patients had their first peritonitis episode within 6 months (Pulliam et al. 2014). A recently published study reported that the first peritonitis episode can change peritoneal membrane function. Several studies have already been conducted to assess the impact of early onset peritonitis (EOP) on outcomes in PD patients. EOP has not been defined consistently, with definitions varying from 3 to 24 months after PD commencement (Fourtounas et al. 2006; Harel et al. 2006; Hsieh et al. 2014). Additionally, previous results were not convincing due to the absence of significant findings in and relatively small sample sizes of these studies (Fourtounas et al. 2006; Harel et al. 2006).

In summary, the definition of early onset peritonitis remains controversial. Furthermore, the impact of early onset peritonitis on the prognosis of PD patients is still without conclusive evidence. In this study, we defined peritonitis occurring within 6 months after PD initiation as EOP. We aimed to compare the prognosis of EOP and non-EOP peritoneal dialysis patients.

## Subjects and methods

### Patients

This was a retrospective study including all patients in our unit who initiated PD between January 1, 2004, and July 31, 2013. All patient outcomes were followed-up up until July 30, 2014. All patients had double cuff silastic PD catheters placed using sterile surgical techniques. Patient demographics, etiology of ESRD and PD duration were obtained by review of patient charts and the computerized database in our unit. Patients were followed until transfer to hemodialysis, renal transplantation or death. Death during PD or within 1 month after conversion to HD was regarded as PD-related mortality. Clinical outcomes were mortality and technical failure. Patients who transferred to HD were censored from patient survival analysis, while patients who died were censored from analysis of technique failure. Exclusion criteria were as follows: (1) PD duration of less than 3 months, (2) inadequate clinical follow-up information, (3) renal transplantation, and (4) prior history of hemodialysis. The study protocol was approved by the ethics committee of our institution.

### Diagnosis of early onset peritonitis

Peritonitis was diagnosed in accordance with published guidelines from the International Society of Peritoneal Dialysis and according to the following standard criteria: clinical signs of peritoneal inflammation, positive culture of peritoneal fluid, and cloudy dialysate with an elevated dialysate white blood cell count of more than 100/mm^3^ (Li et al. 2010). Early onset peritonitis was defined as peritonitis occurring within 6 months of PD initiation.

### Treatment of peritonitis

All patients were assessed by PD unit/renal ward nurses and reviewed by a physician at diagnosis. Empiric treatment consisted of intraperitoneal cefathiamidine (2 g/day) and etimicin (200 mg/day). Antibiotic treatment was tailored once antimicrobial sensitivities were available. The standard duration of antibiotic treatment was 2 weeks. Treatment for longer than 2 weeks was left to the discretion of the physician. PD catheters were removed and patients were switched to hemodialysis if they demonstrated a lack of improvement within the first week of appropriate antibiotic therapy or culture results indicated fungal infection.

### Collecting of clinical characteristics

Data for all subjects during their following up period, including age; gender; serum albumin, creatinine, calcium, and phosphate levels; KT/V; and residual renal function were collected from our center. All peritonitis episodes were recorded, and for each peritonitis episode, the causative microorganism was recorded, if isolated.

### Statistical analysis

**Fig. 1:**
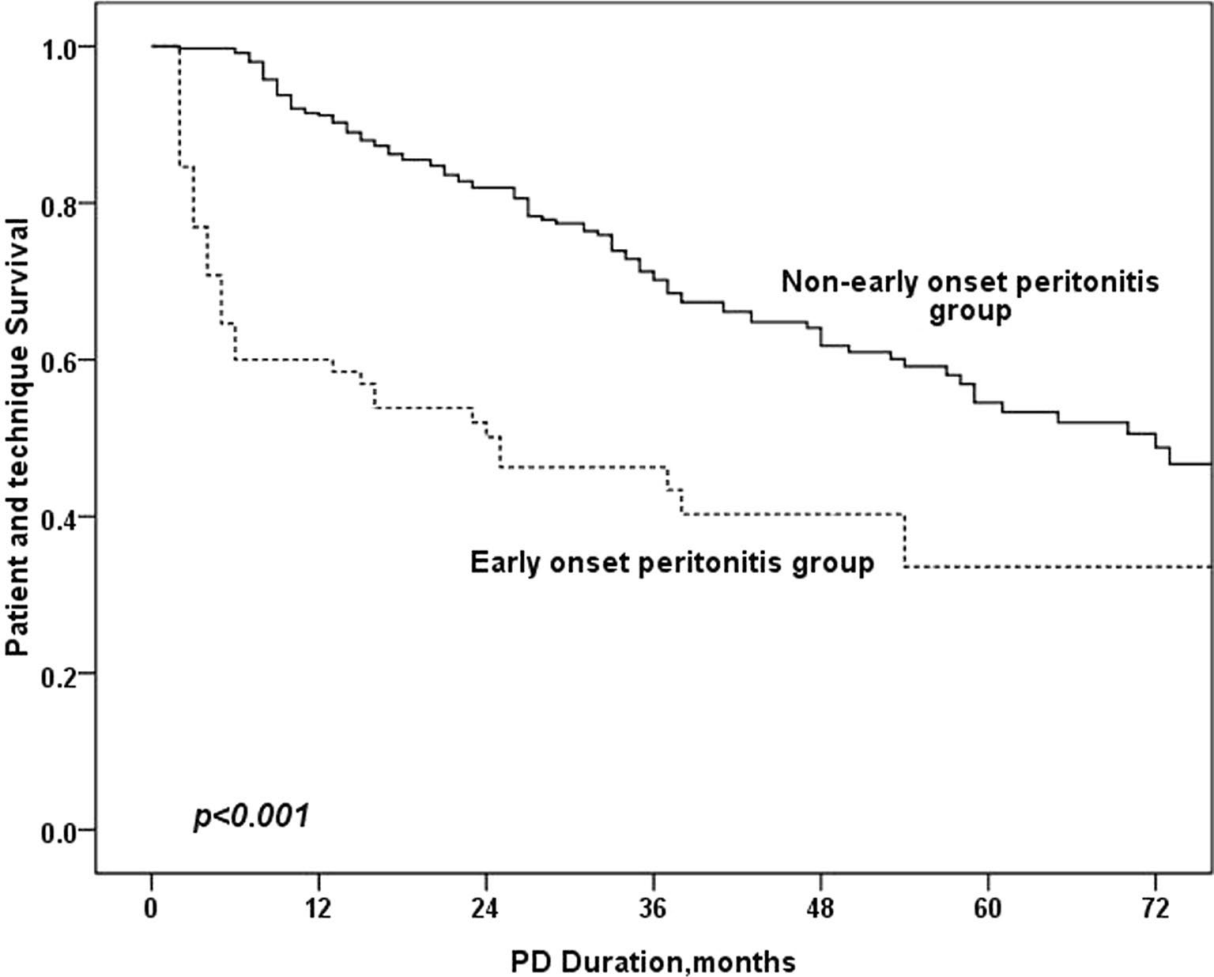
Patient and technique survival in EOP and non-EOP group

**Fig. 2:**
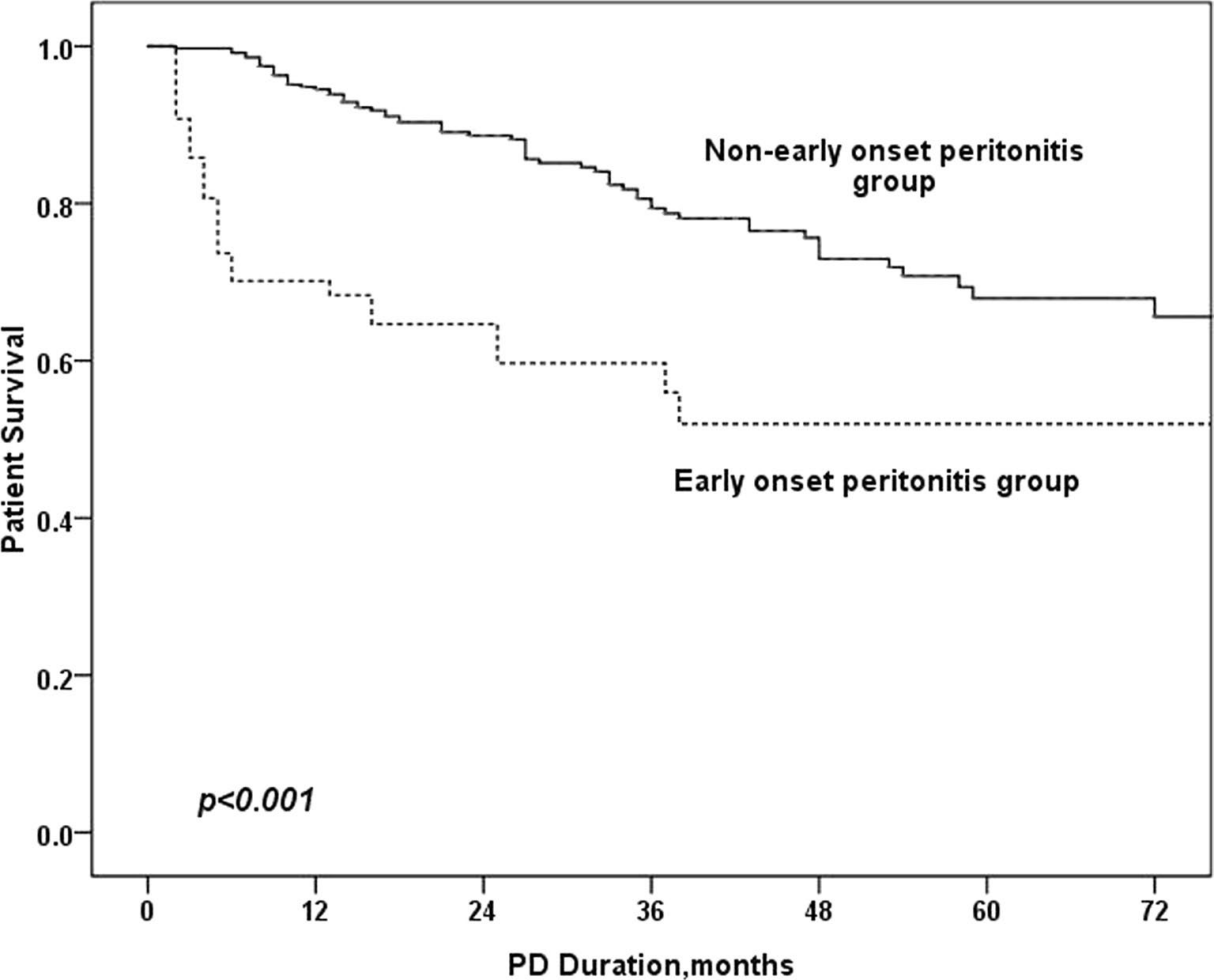
Patient survival in EOP and non-EOP group

**Fig. 3:**
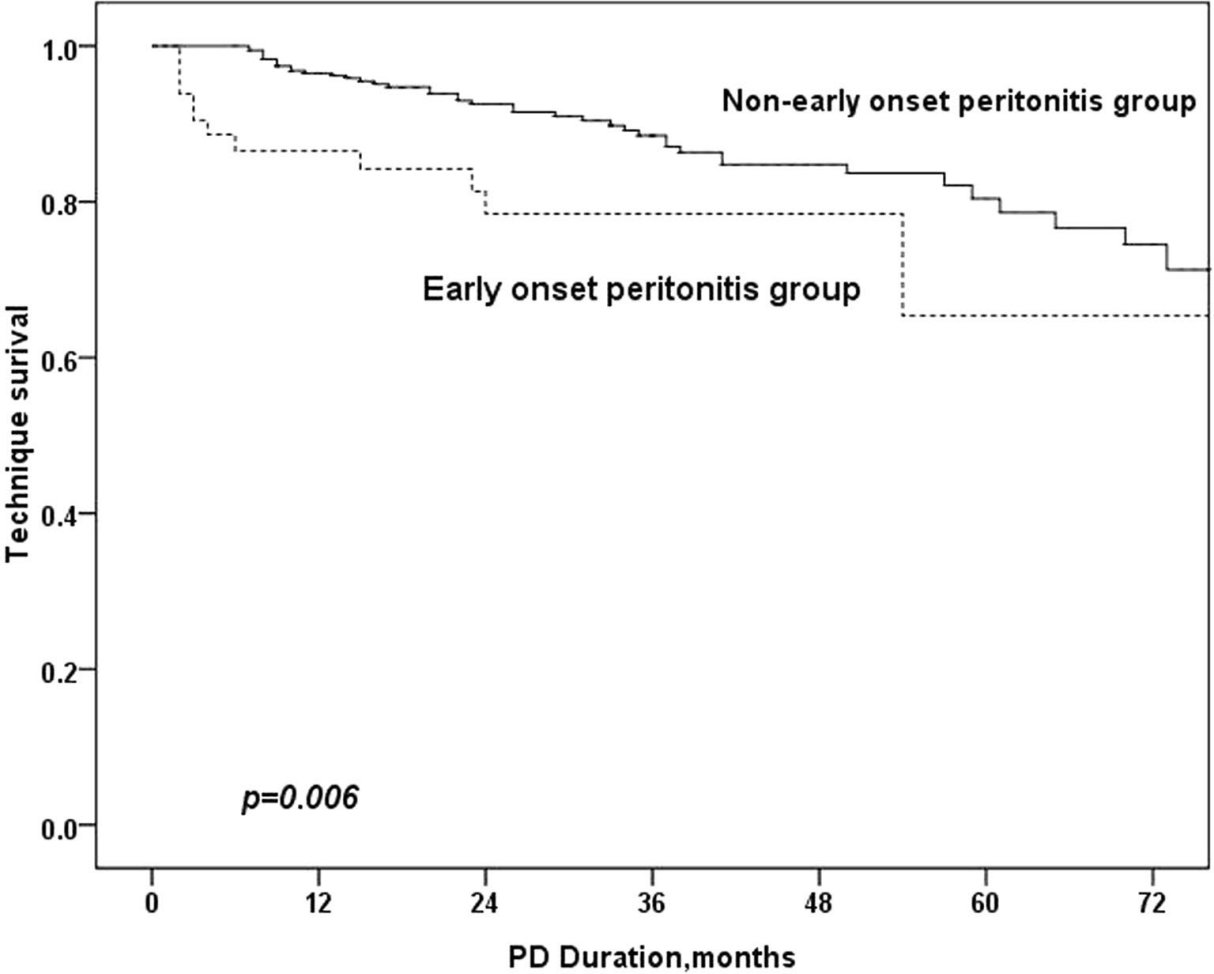
Technique survival in EOP and non-EOP group

Continuous variables are presented as the mean ± SD, and categorical variables are expressed as percentages unless otherwise stated. For comparisons of continuous variables between two groups, Student’s *t* test was used. Correlations were tested using the Pearson correlation method. The Kolmogorov–Smirnov test was used to analyze the distribution of continuous data for the presence of a normal distribution. Relationships between 2 or more groups of data were analyzed using the Pearson Chi square test. Survival curves were generated using the Kaplan–Meier method and compared using the log-rank test. Factors predictive of patient and technique survival were identified using Cox regression analyses. Factors with *p* < 0.10 in univariate analyses were entered into a multivariate Cox regression model. A backward elimination procedure with a removal criterion of *p* > 0.05 was performed to identify independent predictors of patient and technique survival. All computations were performed using SPSS 17.0 for Windows (SPSS Inc, Chicago, IL, USA), and p < 0.05 was considered statistically significant (Figs. 1, 2, 3).

## Results

During the study period, 474 subjects were referred to the dialysis center. Fourteen patients underwent renal transplantation. Twenty-one subjects died within 3 months of PD initiation. Twenty-eight patients were transferred out of the unit, and the other three patients exhibited renal function recovery. Thus, 189 patients had at least one episode of peritonitis.

### Patient characteristics

Of the study subjects, 43.9 % were female (n = 83) and 56.1 % were male (n = 106). The mean age of the subjects was 57.5 ± 15.9 years. The mean duration of treatment was 32.4 ± 23.1 months (range 3–88 months). Additional demographic characteristics, etiology of ESRD, comorbid conditions and laboratory characteristics of the patients are shown in Table 1.

**Table 1:** Comparison of characteristics in EOP and non-EOP patients

Clinical parameters	Non-EOP (n = 134)	EOP (n = 55)	*p* value
Age (years)	56.9 ± 15.8	60.8 ± 16.1	0.092
Gender (male/female)	201/151	29/26	0.543
BMI (kg/m^2^)	22.8 ± 3.7	23.1 ± 4.3	0.361
Diabetes [n (%)]	83 (23.6)	14 (25.5)	0.761
Etiology of ESRD [n (%)]			0.712
Primary glomerulonephritis	81 (60.4)	29 (52.7)	
Diabetic nephropathy	21 (15.7)	8 (14.5)	
Hypertensive nephropathy	15 (11.2)	9 (16.4)	
Other	17 (12.7)	9 (16.4)	
Serum albumin (g/dl)	3.1 ± 0.6	2.8 ± 0.6	0.002
Hemoglobin (g/dl)	10.4 ± 2.0	10.6 ± 2.1	0.576
Phosphorus (mmol/l)	1.57 ± 0.46	1.45 ± 0.38	0.08
Calcium (mmol/l)	2.11 ± 0.25	2.08 ± 0.19	0.434
CRP	4 (4,13)	4 (4,21)	0.632
KT/V urea	1.92 ± 0.51	1.87 ± 0.29	0.603
RRF (ml/min/1.73 m^2^)	0.62 ± 0.41	0.67 ± 0.46	0.521

*EOP* early onset peritonitis, *BMI* body mass index, *CRP* C-reactive protein, *RRF* residual renal function

### Organisms causing peritonitis in EOP and non-EOP patients

In total, 271 peritonitis episodes occurred in 189 patients, and the peritonitis rate was 42.1 episodes per patient-month. The mean peritonitis-free period was 22 ± 15 months. During the study period, 69 (36.5 %) patients had more than 1 episode of peritonitis. Fifty-five (29.1 %) patients were diagnosed with EOP. Peritonitis episodes occurred more frequently in EOP patients (28.7 episodes per patient-month) than in non-EOP patients (49.4 episodes per patient-month).

The culture positive rate was 80.3 %. Comparisons of the culture results between the two groups are shown in Table 2. The organisms causing peritonitis did not differ significantly between the two groups.

**Table 2:** Comparison of orgnisms in causing first peritonitis in EOP and non-EOP patients

Clinical parameters	EOP (n = 55)	Non-EOP (n = 134)	*p* value
Gram-positive organisms	30	75	0.915
Staphylococcus aureus	14	40	
Coagulase-negative staphylococcus	8	18	
Streptococcus species	3	7	
Enterococcus species	3	6	
Other gram-positives	2	4	
Gram-negative organisms	11	35	0.447
Escherichia coli	4	13	
Klebsiella species	4	10	
Serratia species	1	5	
Acinetobacter species	1	4	
Other gram-negatives	1	3	
Fungi	1	3	0.995
Culture negative	13	21	0.344

*EOP* early onset peritonitis

### Causes of death and technique failure

In total, 59 and 84 subjects died in the non-EOP and EOP groups, respectively. Twenty-nine non-EOP and 44 EOP patients died as a result of cardiovascular events; these events included cardiac arrest (n = 7 and n = 8 in the non-EOP and EOP groups), acute myocardial infarction (n = 6), cardiac arrhythmias (n = 5), heart failure (n = 5 and n = 11 in the non-EOP and EOP groups), and stroke (n = 6 and n = 16 in the non-EOP and EOP groups). Fifteen subjects died of infection, of whom 6 died of peritonitis, 6 died of pneumonia, and 3 died of sepsis. The other 15 subjects died of cachexia (n = 5), gastrointestinal bleeding (n = 3), malignancy (n = 3) and unknown reasons (n = 4). Thirty-six subjects were transferred to hemodialysis. The most common cause for this transfer was peritonitis (n = 16), including refractory peritonitis (n = 6), recurrent peritonitis (6) and fungal peritonitis (n = 4). Other causes included ultrafiltration failure (n = 10), refractory heart failure (n = 8) and tunnel infection (n = 3).

### Comparison of outcome in EOP and non-EOP groups

As is shown in Table 3, age, comorbid diabetes mellitus, serum albumin level, CRP, RRF and EOP were univariately associated with mortality in PD patients. In the multivariate Cox regression model, EOP was an independent risk factor for patient mortality (HR 2.03, RI 1.09–3.80, p = 0.026), technique failure (HR 1.69, RI 1.12–2.87, p = 0.015) and total survival (HR 1.73, RI 1.12–2.68, p = 0.013).

**Table 3:** Predictors of mortality and technique failure in PD patients

Variables	Univariate Cox regression analysis			Multivariate Cox regression analysis		
	B	HR (95 % CI)	*p* value	B	HR (95 % CI)	*p* value
Predictors of mortality and technique failure in PD patients						
Age (per 10-year increase)	0.323	1.38 (1.22–1.56)	<0.001	0.201	1.22 (1.07–1.39)	0.002
Albumin (per 1 g/dl decrease)	0.993	2.70 (2.02–3.61)	<0.001	0.744	2.10 (1.53–2.90)	<0.001
Diabetes mellitus	0.709	2.03 (1.42–2.91)	<0.001	0.414	1.51 (1.03–2.10)	0.038
Log CRP	1.071	2.87 (2.18–4.51)	<0.001	0.819	2.44 (1.82–3.89)	<0.001
RRF (per 1 ml/min × 1.73 m^2^ decrease)	0.568	1.68 (1.31–2.75)	0.003	0.497	1.41 (1.12–2.07)	0.024
EOP group	750	2.12 (1.39–3.24)	<0.001	0.550	1.73 (1.12–2.68)	0.013
Predictors of technique failure in PD patients						
Albumin (per 1 g/dl decrease)	0.609	1.84 (1.12–3.03)	0.010	0.826	1.92 (1.32–2.81)	0.016
Diabetes mellitus	0.746	2.11 (1.15–3.88)	0.017	0.780	2.19 (1.10–4.35)	0.025
RRF (per 1 ml/min × 1.73 m^2^ decrease)	0.913	2.71 (1.88–3.79)	<0.001	0.887	2.64 (1.71–3.65)	0.004
EOP group	0.805	2.24 (1.62–3.86)	0.006	0.710	2.03 (1.09–3.80)	0.026
Predictors of mortality in PD patients						
Age (per 10-year increase)	0.597	1.82 (1.53–2.16)	<0.001	0.454	1.58 (1.31–1.90)	<0.001
Albumin (per 1 g/dl decrease)	1.29	3.62 (2.54–5.16)	<0.001	0.915	2.50 (1.68–3.73)	<0.001
Diabetes mellitus	0.95	2.59 (1.67–4.00)	<0.001	0.517	1.68 (1.08–2.61)	0.022
Log CRP	1.31	3.84 (2.16–5.63)	<0.001	0.926	2.89 (1.92–4.86)	<0.001
RRF (per 1 ml/min × 1.73 m^2^ decrease)	0.432	1.57 (1.05–2.41)	0.028	–	–	–
EOP group	0.865	2.37 (1.42–3.97)	<0.001	0.510	1.69 (1.12–2.87)	0.015

*HR* hazard ratio, *CI* confidence interval, *CRP* C-reactive protein, *RRF* residual renal function, *EOP* early onset peritonitis

## Discussion

In this study, we determined that EOP occurred in approximately one-third of peritonitis patients. We also confirmed that EOP was an independent risk factor for poorer outcomes in PD patients.

The definition of early onset peritonitis remains controversial. The BRAZPD study revealed that the median time to first peritonitis episode in elderly PD patients was 6 months (Martin et al. 2011). In a recently published study of 1677 incident peritoneal dialysis patients in America, three-fourths of patients exhibited a first peritonitis episode within the first 6 months of peritoneal dialysis treatment (Pulliam et al. 2014). In our study, one-third of peritonitis episodes occurred during the first 6 months after PD initiation. Based on these observations, it is reasonable to use 6 months as the cut-off point to define early peritonitis.

In this study, we found that gram-positive and gram-negative bacteria were the causative organisms in 54.5 and 23.8 % of peritonitis cases, respectively. The most common bacteria causing the first peritonitis episode was *Staphylococcus aureus*. This result is in accordance with research conducted by Fourtounas et al. (2006), Hsieh et al. (2014). The organisms implicated in causing EOP and late onset peritonitis did not differ significantly (Table 2). However, peritonitis rates were higher in EOP patients than in patients with late onset peritonitis. This may because of unskilled manipulation after PD (Fourtounas et al. 2006).

This research found that EOP was an independent risk factor for poorer outcomes in PD patients. This result is in accordance with previous studies on this topic (Isla et al. 2014; Kavanagh et al. 2004; Li and Chow 2013). There are several explanations for this result. First, peritonitis has been confirmed to be an independent risk factor for poor outcomes in PD patients. In this study, patients with EOP had increased peritonitis rates. Fourtounas et al. (2006) also reported this phenomenon. Moreover, studies have reported that peritonitis can alter natural peritoneal membrane characteristics and cause long-lasting alterations in peritoneal transport states (Radtke et al. 2004; van Diepen et al. 2014), which may result in poor outcomes. Second, patients with EOP may have poor nutritional status. In this study, compared to non-EOP patients, EOP patients were older and had a lower ALB level, both of which may negatively impact patient outcomes.

We defined EOP as peritonitis occurring within 6 months after PD initiation. This is different from research conducted by Hsieh et al. (Fourtounas et al. 2006; Hsieh et al. 2014). In their study, using the median duration to peritonitis, they defined peritonitis occurring within 24 months as EOP. Thus, their definition may not be generalizable to other patient populations, affecting study results. In this study, approximately 30 % of subjects were diagnosed with EOP. This result is in accordance with research conducted by Martin et al. (2011) and Pulliam et al. (2014), supporting our definition of EOP. Furthermore, early diagnosis of EOP may be associated with earlier intervention and, therefore, improved prognosis.

In this study, we also demonstrated that older age, lower albumin level, diabetes diagnosis and residual renal function were risk factors for patient mortality and technique failure. These risk factors have already been well established in several large prospective studies (Collins et al. 1999; Vonesh and Moran 1999; Wang et al. 2004).

There are several limitations to our study. First, a limitation of this study is that it was conducted in a single center. Second, due to the retrospective nature of this study, some potentially important characteristics, such as literacy and SGA and comorbidity index scores, were not evaluated.

## Conclusions

In conclusion, our study demonstrates that EOP has a negative effect on outcomes in PD patients. To confirm this relationship, clarify its underlying mechanisms, and identify risk factors for EOP in CAPD patients, a prospective study needs to be conducted.
